# Synthetic and Natural Red Food Dyes Affect Oxidative Metabolism and the Redox State in the Nauplii of Brine Shrimp *Artemia franciscana*

**DOI:** 10.3390/antiox14060634

**Published:** 2025-05-25

**Authors:** Gianluca Fasciolo, Gaetana Napolitano, Maria Teresa Muscari Tomajoli, Eugenio Geremia, Adriana Petito, Carlos Gravato, Andreia C. M. Rodrigues, Ana L. Patrício Silva, Chiara Maria Motta, Claudio Agnisola, Paola Venditti

**Affiliations:** 1 Department of Biology, Federico II University of Naples, Complesso Universitario Monte Sant’Angelo, Via Cinthia, 80126 Naples, Italy; gianluca.fasciolo@unina.it (G.F.); adriana.petito@unina.it (A.P.); mottacm@unina.it (C.M.M.); agnisola@unina.it (C.A.); 2 International Ph.D. Program, UNESCO Chair “Environment, Resources and Sustainable Development”, Department of Science and Technology, Parthenope University of Naples, Centro Direzionale, Isola C4, 80143 Naples, Italy; mariateresa.muscaritomajoli001@studenti.uniparthenope.it (M.T.M.T.); eugenio.geremia@studenti.uniparthenope.it (E.G.); 3 Departamento de Biologia Animal, Faculdade de Ciências da Universidade de Lisboa (FCUL), Campo Grande, 1749-016 Lisboa, Portugal; cagravato@fc.ul.pt; 4 Centre for Ecology, Evolution and Environmental Changes (cE3c) & CHANGE–Global Change and Sustainability Institute, Faculdade de Ciências da Universidade de Lisboa FCUL, Campo Grande, 1749-016 Lisboa, Portugal; 5 Centro de Estudos de Ambiente e do Mar (CESAM), Department of Biology, University of Aveiro, 3810-193 Aveiro, Portugal; rodrigues.a@ua.pt (A.C.M.R.); ana.luisa.silva@ua.pt (A.L.P.S.)

**Keywords:** E120, E124, ROS production, oxidative damage, antioxidant enzymes, antioxidant capacity, redox homeostasis

## Abstract

The food industry widely uses dyes from animal and plant sources, but their discharge into water bodies can harm aquatic animals. Red food dyes increase reactive oxygen species (ROS) production, disrupting redox homeostasis in *Artemia franciscana* nauplii, although the underlying mechanisms are unclear. In this study, we exposed *Artemia franciscana* cysts for 48 h to three different red dyes: E124 (synthetic), E120 (animal-based) or Vegan red (plant-based) and evaluated the oxidative metabolism and redox status in the hatched nauplii. Only E120 and VEG increased oxygen consumption. E124 and VEG increased mitochondrial Complex I activity, while all dyes enhanced the activity of Complex III. The levels of reactive oxygen species (ROS) and NADPH oxidase activity were increased by all red dyes. E120 and E124 increased antioxidant enzyme activity to a greater extent than VEG. Additionally, only E120 and E124 increased total antioxidant capacity. Nevertheless, E124 exposure induced redox imbalance (increased lipid and protein oxidative damage). Our data, as a whole, allow us to conclude that red dyes can influence the oxidative capacity and redox state of *Artemia franciscana* nauplii with more harmful effects in the presence of E124, thus drawing attention to their potentially severe influence on aquatic life.

## 1. Introduction

Dyes are widely used in the food industry to improve the attractiveness and aesthetic appearance of foods for end users, and a significant amount of them is discharged into the aquatic environment during the production processes [1].

Synthetic and natural (animal and vegetal-based bio-colour) dyes have presented a potential danger for aquatic biota [2,3] through indirect or direct actions. Indirect action depends on the colouration of the water, which, by decreasing the amount of sunlight that passes through the water column, reduces oxygen availability due to compromised photosynthesis [4]. Direct action can depend on the presence of aromatic centers (azo dyes) in the molecular structure of most artificial dyes, whose metabolism and degradation produce carcinogenic and mutagenic molecules such as anilines, benzidines, and benzene, which can induce cell death [5]. Toxicity effects related to azo dyes have been reported for some freshwater species, including the microcrustacean *Daphnia magna* [6], the cnidarian *Hydra attenuata* [7], and the fish *Labeo rohita* [8]. We have previously reported that *Artemia franciscana* nauplii hatched in the presence of E120 show an altered phototactic response [2]. Dangerous by-products can also be derived from natural dyes of vegetal origin [9]. For example, the mushroom-extracted dye erythrostominone impairs the development and behaviour of zebrafish in the early stages of development [10].

So far, information concerning the mechanisms by which food dyes affect the life of aquatic organisms is scarce. Energetic metabolism is crucial in animal physiological and behavioural functions. Therefore, analysing biomarkers linked to energy balance has high ecological relevance since detoxification responses to chemical stressors are energetically costly for exposed organisms, and energy allocation for other physiological processes may be decreased or impaired [11,12]. Indeed, Abe et al. [12] found lower energy expenditure accompanied by unaltered levels of metabolic substrates and reduced swimming activity in zebrafish larvae exposed to environmental stress. Little is known about dyes’ effects on metabolism and the production and management of oxidative metabolism by-products, such as reactive oxygen species (ROS) [13]. Environmental contaminants increase the production of ROS, such as superoxide (O_2_^−^), hydroxyl radicals (OH^+^) and hydrogen peroxide (H_2_O_2_) [14], which can trigger oxidative damage in aquatic animals. Both natural and synthetic dyes induce oxidative damage and increase the activity of glutathione-s-transferase, an enzyme of phase II detoxification of xenobiotics [15]. Furthermore, artificial or natural food dyes increase ROS content in the nauplii of *Artemia franciscana* and reduce susceptibility to oxidants in *Danio rerio* embryos [16], drawing attention to food dyes’ effect on animal redox homeostasis.

To gain a better understanding of the mechanisms underlying the responses triggered by sublethal concentrations of synthetic and natural dyes in *Artemia franciscana* nauplii, we compared the effects of the synthetic dye Ponceau Red (E124), the natural, commercial bio-colour from crushed radish, black currant and apple extracts (Red Vegan, VEG) and the natural dye Cochineal Red (E120) extracted from the insects *Dactylopius coccus* and *Kermes vermilio* (in Table 1, the main red molecules in each dye used are reported). We evaluated parameters related to the respiratory capacity (in vitro oxygen consumption and mitochondrial complexes activity) and redox state (ROS content, NADPH oxidase activity, lipid and protein oxidative damage markers and antioxidant capacity).

## 2. Materials and Methods

### 2.1. Preparation of Artemia franciscana Nauplii

Artemia cysts were purchased from a commercial supplier (Hobby^®^, Gelsdorf, Germany). Fixed amounts (0.5 g) of *Artemia franciscana* cysts were diluted in 500 mL of artificial seawater (ASW) for the control group (C) or ASW plus 1.2 g L^−1^ of one of the three red food dyes for the VEGR, E120 or E124 groups. The environmental conditions were a temperature of 25 °C, continuous aeration, an artificial photoperiod (16/8 h light/dark) and water salinity of 36‰ according to the specific requirements for *Artemia franciscana* [18]. We chose dyes used in the food industry and for homemade foods. E124 and E120 were aqueous solutions (20% dyes), while VEG was a powder containing extracts of radish, blackcurrant and apple, plus unknown quantities of maltodextrin and citric acid. Since we did not have information on the environmental concentrations of the red dyes we were testing, we decided to use the concentration suggested by the manufacturer for homemade foods. Furthermore, to our knowledge, there is no information on the concentrations of dyes in effluents from the food industry, and only some information is available on their concentrations in textile industry wastewater [19]. The reported values are very variable (from 10 to 50 mg∙L^−1^ up to 7000 mg∙L^−1^), which probably reflects the different environmental and political conditions of the countries where the industries were located. Therefore, we decided to use a relatively high dye concentration, since our study aimed to obtain a positive control to test the mechanisms with which the experimental model reacts to stress imposed by red dye pollution. The nauplii hatched within a few hours of incubation, and we collected them after 48 h. In all groups, we never found a mortality higher than 10% [2,20]. After collecting, nauplii were resuspended in saline solution (220 mM mannitol, 70 mM sucrose, 1 mM EDTA, 10 mM Tris, pH 7.4), maintained at 0 °C and homogenised using a glass Potter–Elvehjem homogenizer set at the standard speed (500 rpm) for 1 min. The Bio-Rad Bradford colorimetric assay (Bio-Rad, Hercules, CA, USA) was used to evaluate the protein concentration in the homogenates. Some aliquots of homogenates were used for oxygen consumption determinations, while others were immediately frozen and stored at −80 °C for subsequent analyses.

### 2.2. Oxygen Consumption

The oxygen consumption rates in homogenates of nauplii were polarographically determined at 25 °C using a Hansatech oximeter (Oxygraph^+^, Hansatech Instruments Ltd., Norfolk, UK). To 1.0 mL of incubation medium (145 mM KCl, 30 mM Hepes, 5 mM KH_2_PO_4_, 3 mM MgCl_2_, 0.1 mM EGTA, pH 7.4), 0.2 mg of homogenate protein was added. After instrument stabilisation, Succinate (10 mM) plus 5 μM rotenone or pyruvate plus malate (10 and 2.5 mM, respectively) were used as respiratory substrates. The measures were carried out without (State 4) or with (State 3) ADP (500 μM). The respiration rates were reported as nmol O min^−1^∙mg^−1^ protein. The ratio between the oxygen consumption rates in State 3 and State 4 furnishes the respiratory control ratio (RCR), an index of the coupling between oxidative phosphorylation and ATP synthesis.

### 2.3. Mitochondrial Complexes Activities

The activities of complexes I, II, and III of the mitochondrial electron transport chain were measured by spectrophotometric methods [21,22] (Synergy™ HTX Multi-Mode Microplate Reader, BioTek, Ahmedabad, India).

Complex I activity was expressed as μmol NADH/min∙mg protein. Complex II activity was expressed as μmol 2, 6-dichlorophenylindophenol (DCPIP)/min∙mg protein. Complex III activity was expressed as μmol cytochrome c reduced/min∙mg protein.

Cytochrome c oxidase (COX) activity, the fourth complex of the mitochondrial electron transport chain, was evaluated through a polarographic procedure [22] (Hansatech oximeter, Oxygraph^+^, Hansatech Instruments Ltd., Norfolk, UK). COX activity was reported as µmol O/min∙mg protein.

### 2.4. Total ROS Level and NADPH Oxidase (NOX) Activity

ROS content was detected following the increase in dichlorofluorescein (DCF, fluorescent compound) obtained by the oxidation of 2′,7′-dichlorodihydrofluorescin diacetate (DCFH-DA, non-fluorescent compound) due to the ROS present in samples [23]. DCF fluorescence was measured using a Synergy fluorescence spectrophotometer (λ_Ex_ 485 nm and λ_Em_ nm). Parallel blanks were used to correct background fluorescence (conversion of DCFH to DCF in the absence of homogenate). ROS content was reported as Relative Fluorescence Units (RFUs)/μg protein.

NOX activity was assayed using a spectrophotometric method (550 nm) [22] by evaluating the reduction of ferricytochrome c acetylated (80 μM) in the presence of NADPH (1 mM) at room temperature, induced by 0.2 mg of homogenate protein. NOX activity was calculated by the difference in the measures performed in the presence and absence of 100 μg/mL superoxide dismutase and expressed as µmol Ac cyt c/mg protein.

### 2.5. Activities of the Antioxidant Enzymes (GPX, GR, Catalase and SOD) and Total Antioxidant Capacities (TACs)

The glutathione peroxidase (GPX) activity of 0.02 mg of nauplii homogenate protein was assayed at 25 °C by following the rate of NADPH oxidation in the presence of H_2_O_2_ as a substrate, GSH, and GR [24].

Glutathione reductase (GR) activity of 0.02 mg of nauplii homogenate protein was assayed at 25 °C by measuring the NADPH oxidation rate after adding GSSG [25].

In both assays, the NADPH oxidation rate was followed at 340 nm using a multi-mode microplate reader (Synergy™ HTX Multi-Mode Microplate Reader, BioTek), and the measures were expressed as µmol NADPH/min∙mg protein.

Catalase (CAT) activity was spectrophotometrically assayed on 0.02 mg of homogenate protein with 5 mM H_2_O_2_ after the lysis of the samples obtained with 1% Triton [26]. The enzyme activity was reported as μmol H_2_O_2_/min∙mg protein.

Superoxide dismutase specific activity was carried out spectrophotometrically (550 nm) at 25 °C, by measuring the decrease in the reduction rate of cytochrome c induced by the superoxide radicals generated by the system xanthine–xanthine oxidase. Homogenate samples (0.05 mg of protein) treated with 0.2% Triton were added to a buffer solution containing 0.1 mM EDTA, 2 mM KCN, 50 mM KH_2_PO_4_, 2 μM cytochrome c and 50 μM xanthine with a pH of 7.8. In total, 0.03 U of xanthine oxidase was added to start the reaction. One unit (U) of SOD activity is defined as the concentration of enzyme that inhibits cytochrome c reduction by 50% in the presence of xanthine + xanthine oxidase [22]. The enzyme activity was reported as U/mg protein.

The total antioxidant capacity (TAC) of aliquots of homogenate containing 0.01 mg of protein was assessed at 734 nm using a decolorization assay according to Erel [27], with modifications [21]. The pre-formed radical monocation of 2,2′-azinobis-(3-ethylbenzothiazoline-6-sulfonic acid) (ABTS^•+^) is generated by oxidation of ABTS (7 mM) with potassium persulfate (245 mM), and the cation radical is decolorized by antioxidants of *Artemia franciscana* homogenates according to their concentrations and antioxidant capacities. A stock solution of 3,5-Di-tert-4-butylhydroxytoluene (BHT) at 2.5 mM was used for the calibration curve. Total antioxidant capacity was expressed as BHT Equivalents/mg protein.

### 2.6. Lipid and Protein Oxidative Damage

The extent of lipid peroxidative processes in homogenates of nauplii was evaluated by measuring the lipid hydroperoxide (HP) levels in 10 µg of homogenate protein, diluted in 0.2 mM EDTA and 0.124 M Tris-HCl buffer at a pH of 7.6 [28]. NADPH (0.2 mM) absorbance reduction, determined by the coupling of reactions catalysed by glutathione peroxidase (0.025 U/mL) and glutathione reductase (0.025 U/mL) enzymes in the presence of 0.425 mM GSH, followed at 340 nm. HP levels were reported as nmol NADPH/min∙mg protein. Lipid peroxidation was also assayed by measuring the levels of Thiobarbituric Acid Reactive Substances (TBARs) according to the method of Okhawa [29], adapted to microplate [30]. To 200 μL of homogenate, 4 µL of 4% antioxidant butylated hydroxytoluene (BHT) was added to avoid endogenous oxidation of lipids. The content of TBARs was determined at 535 nm and expressed in pmol MDA/mg protein using an extinction coefficient (ε) of 15,900 M^−1^ cm^−1^.

The extension of oxidative damage to proteins was determined by measuring the level of protein-bound carbonyls (CO). CO content was quantified by their reaction with 2,4-dinitrophenyl hydrazine (DNPH) using a simplified method adapted to a Nunc standard 96-well plate [22,31]. Protein carbonyls (CO) were calculated using the extinction coefficient of DNPH at 450 nm (ε = 22,308 M^−1^ cm^−1^) and an optical path length of 0.1 cm and expressed as µmol CO/mg protein. A multimodal microplate reader (Synergy™ HTX Multi-Mode Microplate Reader, BioTek) was used for all assays.

### 2.7. Data Analysis

GraphPad Prism software, version 9.0 (GraphPad Software Inc., San Diego, CA, USA) was used to evaluate statistical differences. The Shapiro-Wilk and F tests were used to evaluate the data’s normal distribution and equal variance. The significant differences among the experimental groups were determined using one-way analysis of variance (ANOVA), followed by Tukey’s test. In the figure, the mean ± SEM is reported. The *p*-values of * *p* < 0.05, ** *p* < 0.01, *** *p* < 0.001 and **** *p* < 0.0001 were assigned to indicate statistical significance between the different experimental groups.

## 3. Results

### 3.1. Oxygen Consumption

**Figure 1:**
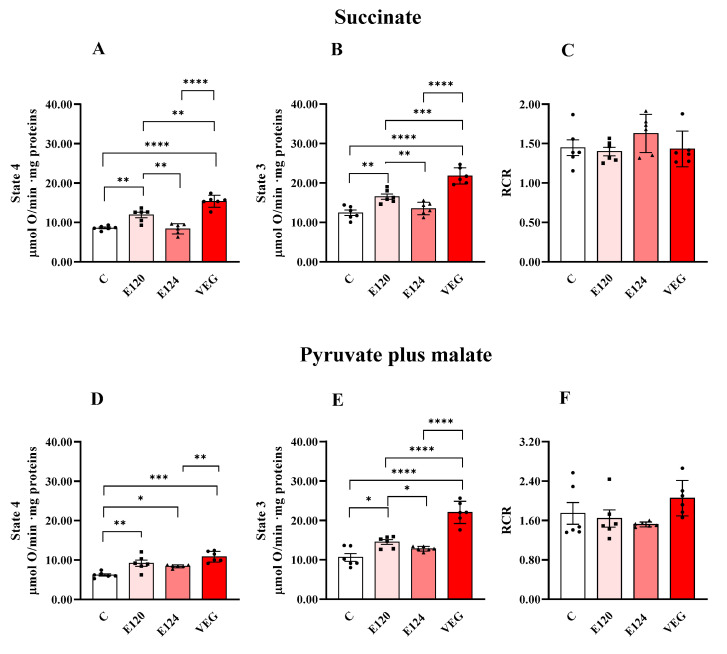
Oxygen consumption in homogenates of *Artemia franciscana* nauplii hatched in the absence (**C**) and presence of E120, E124 and VEG red food dyes. State 3 (in the presence of ADP; Panels (**B**,**E**)), State 4 (in the absence of ADP; Panels (**A**,**D**)) and RCR (Panels (**C**,**F**)) oxygen consumption is detected in presence of succinate (Panels (**A**,**B**)) and pyruvate/malate (Panels (**D**,**E**))-supplemented homogenates. Values are means ± SEMs of six (n = 6) biological replicates. The *p*-values of * *p* < 0.05, ** *p* < 0.01, *** *p* < 0.001 and **** *p* < 0.0001 were assigned to indicate statistical significance between the different experimental groups.

Red food dye treatments affected homogenate oxygen consumption sustained by the respiratory substrates succinate or pyruvate plus malate during both State 4 and State 3 respiration (Figure 1).

E120 and VEG dye exposure increased homogenate oxygen consumption in State 4 and State 3 respiration, independently of the respiratory substrates, compared to the control group. Furthermore, the VEG dye-induced increase was higher than that induced by E120 dye, except that during State 4 respiration, sustained by pyruvate and malate.

Exposure to the artificial red dye E124 did not affect oxygen consumption in the presence of succinate in either State 4 or State 3, compared to the control group, and increased pyruvate-plus-malate-sustained respiration only during State 4. RCR values were unaffected by the treatments.

### 3.2. Mitochondrial Complexes Activities

**Figure 2:**
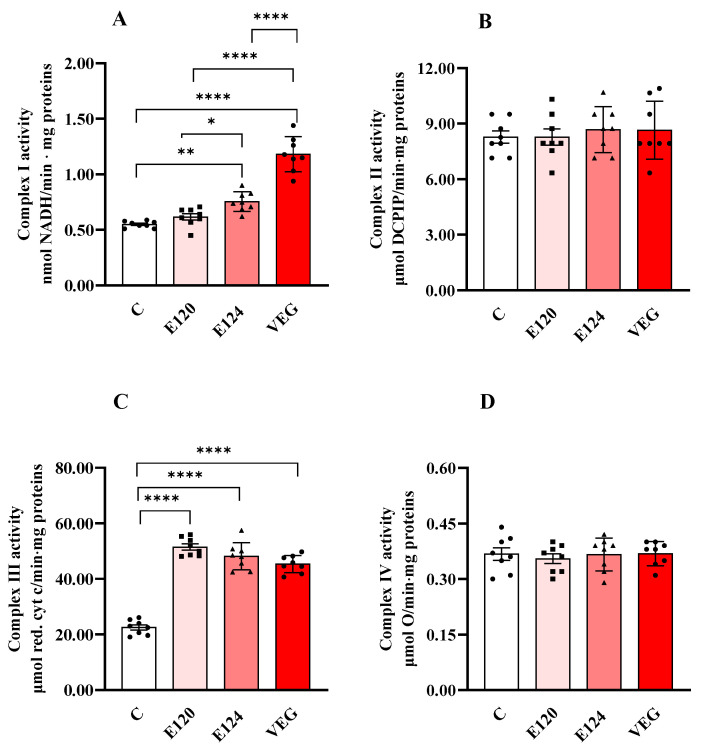
Complex I (Panel (**A**)), Complex II (Panel (**B**)), Complex III (Panel (**C**)) and Complex IV (or cytochrome oxidase, COX, Panel (**D**)) activities in homogenates of *Artemia franciscana* nauplii hatched in the presence of E120, E124 and Vegan red food dyes. Values are means ± SEMs of eight (n = 8) biological replicates. The *p*-values of * *p* < 0.05, ** *p* < 0.01 and **** *p* < 0.0001 were assigned to indicate statistical significance between the different experimental groups.

Treatments with red food dyes induced changes in the activities of respiratory chain complexes I and III (Figure 2A,C), while the activities of complexes II (Figure 2B) and IV (Figure 2D) were not affected by any of the red dyes.

Exposure to E124 and VEG dyes increased Complex I activity (Figure 2A) compared to the control group, with the highest value found in the presence of VEG dye.

All three red food dyes induced a significant and comparable increase in the activity of Complex III (Figure 2C) compared to the control nauplii.

### 3.3. Total ROS Level and NADPH Oxidase (NOX) Activity

**Figure 3:**
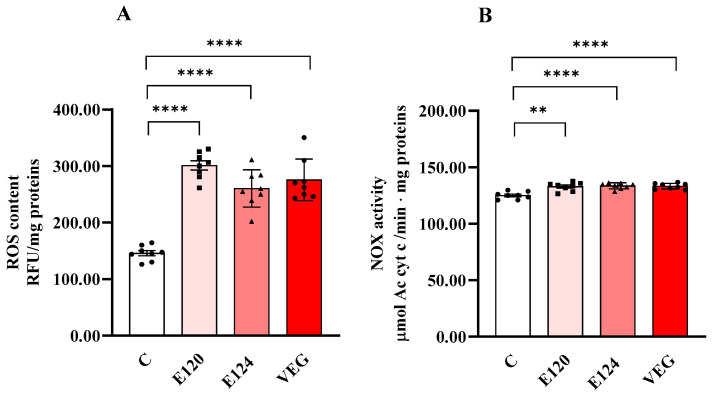
ROS content (Panel (**A**)) and NADPH oxidase (NOX) (Panel (**B**)) activity in homogenates of *Artemia franciscana* nauplii hatched in the presence of E120, E124 and Vegan red food dyes. Values are means ± SEM of eight (n = 8) biological replicates. The *p*-values of ** *p* < 0.01 and **** *p* < 0.0001 were assigned to indicate statistical significance between the different experimental groups.

All red food dyes significantly influenced ROS content (Figure 3A) and NOX activity (Figure 3B) in nauplii.

ROS content significantly increased in the nauplii hatched in the presence of all the red food dyes studied compared to the control group. NOX activity was also slightly but significantly increased by all dyes compared to C.

### 3.4. Antioxidant Enzyme Activity and Total Antioxidant Capacity

**Figure 4:**
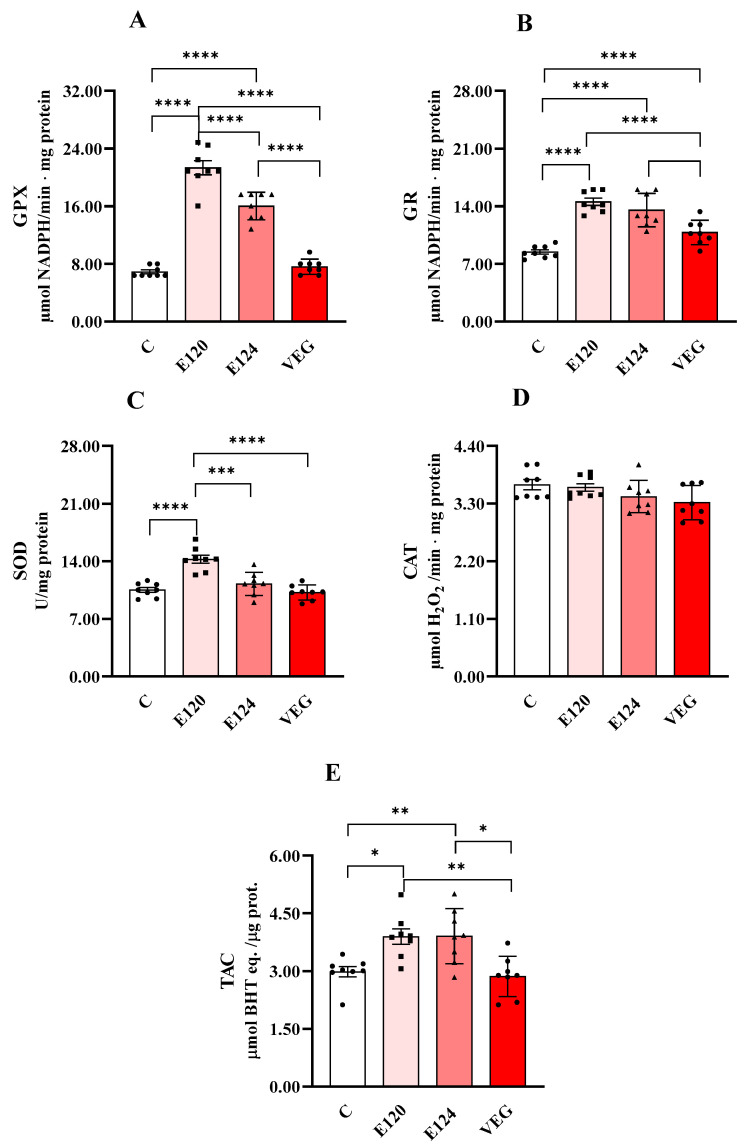
Glutathione peroxidase (GPX, Panel (**A**)), glutathione reductase (GR, Panel (**B**)), catalase (CAT, Panel (**C**)) and superoxide dismutase (SOD, panel (**D**)) activities and total antioxidant capacity (TAC, Panel (**E**)) in homogenates of *Artemia franciscana* nauplii hatched in presence of E120, E124 and Vegan red food dyes. Values are means ± SEMs of eight (n = 8) biological replicates. The *p*-values of * *p* < 0.05, ** *p* < 0.01, *** *p* < 0.001 and **** *p* < 0.0001 were assigned to indicate statistical significance between the different experimental groups.

Figure 4 shows the activities of GPX (Figure 4A), GR (Figure 4B), CAT (Figure 4C) and SOD (Figure 4D) and total antioxidant capacity (TAC, Figure 4E).

Both E120 and E124 significantly increased GPX activity compared to the control group, with a greater effect for E120. GR activity was significantly increased by all dye treatments compared to the control group. Moreover, the effect was lower for VEG with respect to E120 and E124. CAT activity was unmodified by treatments. SOD activity was increased only in the presence of E120 compared to the control group.

Total antioxidant capacity was increased by both E120 and E124 compared to the control group.

### 3.5. Markers of Oxidative Damage to Lipids (Early HPs and Late TBARs) and Proteins (CO)

**Figure 5:**
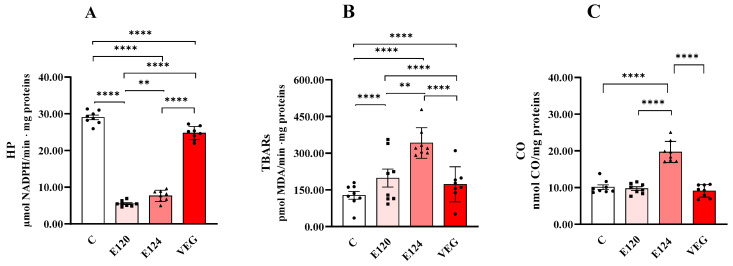
Hydroperoxide (HP; Panel (**A**)), Thiobarbituric Acid Reactive Substance (TBAR; Panel (**B**)) and protein-bound carbonyl content (CO; Panel (**C**)) in homogenates of *Artemia franciscana* nauplii hatched in the presence of E120, E124 and Vegan red food dyes. Values are means ± SEMs of eight (n = 8) biological replicates. The *p*-values of ** *p* < 0.01 and **** *p* < 0.0001 were assigned to indicate statistical significance between the different experimental groups.

All red food dyes significantly affected oxidative damage in terms of HP (Figure 5A), TBAR (Figure 5B) and CO levels (Figure 5C).

Lipid peroxidation is a multi-step process that forms early and late products. HPs are an early product from which molecules such as aldehydes and ketones, including malondialdehyde (MDA), can originate. MDA can react nonspecifically with thiobarbituric acid (TBA), leading to the formation of thiobarbituric acid reactive substances (TBARs), a commonly used but nonspecific indicator of lipid peroxidation.

Dye treatments significantly decreased HP levels compared to the control group. This reduction was greatest for E120 and the least significant for VEG. The order of reduction was E120 > E124 > VEG.

Dye treatments significantly increased TBAR levels compared to the control group. This increase was greatest for E124 and least significant for VEG. The order of increase was E124 > E120 > VEG.

CO levels were significantly increased only in the presence of E124 compared to the control group.

## 4. Discussion

The results of this study demonstrate that the oxidative metabolism of *Artemia franciscana* nauplii is differently affected by the exposure of the cysts to E120, E124, and VEG red food dyes. Specifically, the animal-based E120 and plant-based VEG dyes increased oxygen consumption during States 3 and 4 respiration, whereas E124 had no such effect. These differences in oxygen consumption rates may be attributed to variations in the composition of naupliar populations. Indeed, as previously reported, although the hatching rates observed after 48 h of exposure to E120 and VEG were comparable, exposure to E124 led to a notably higher hatching percentage [32]. Furthermore, in the presence of E124, advanced larval stages were absent 10 days post-hatching [32], suggesting that E124 can interfere with the early stages of nauplii development, which are also crucial for mitochondrial development [33]. Indeed, while in the cysts, mitochondria have no cristae and have low mitochondrial capacity, so hydration of the cysts induces marked biochemical and morphological changes in the mitochondria. Mitochondrial biogenesis proceeds in two stages after hatching. The first one is completed within one hour and is characterised by a rapid increase in the mitochondrial respiratory capacity, cytochrome oxidase, cytochrome b, cytochrome c, and perhaps some morphological changes [33]. In the second stage, there is an increase in the protein-synthesising capacity of the mitochondria as well as striking changes in mitochondrial morphology leading to the formation of cristae. We cannot provide a convincing explanation for the effects of dyes, particularly E124, on the early stages of nauplii development. Still, our data suggest that they influence mitochondrial development differently. Previous studies have shown that 48 h exposure to E120 did not affect naupliar motility [2] and that none of the three dyes significantly altered nauplii in vivo oxygen consumption [16]. The observed increase in State 3 respiration in nauplii exposed to E120 and VEG may represent a compensatory response to dye-induced impairment of haemoglobin-mediated oxygen transport, as suggested by findings that dye pollution can interfere with haemoglobin function and oxygen-carrying capacity in fish [34]. This compensatory response does not appear in nauplii exposed to E124, which probably negatively influences their development in the advanced larval stages.

In E120 and VEG, the increase in State 3 respiration is accompanied by an increase in State 4 oxygen consumption, suggesting an increased proton leakage and subsequent dissipation of proton motive force straddling the inner mitochondrial membrane. However, because the changes in respiratory states are consistent, RCR values do not differ between treatment groups, suggesting that the coupling between electron flow and oxidative phosphorylation is not altered.

Further insights into the metabolic effects of the dyes were obtained by assessing the activities of mitochondrial respiratory complexes in homogenates of nauplii populations hatched after exposure to different dyes. Although these measurements were performed in vitro under non-physiological conditions, such as pH, osmolarity and substrate concentrations, and do not permit evaluation of respiratory coupling, they nonetheless provide valuable quantitative information regarding the maximal catalytic capacity of the individual respiratory chain complexes. Interestingly, the observed increases in State 3 oxygen consumption in E120- and VEG-exposed nauplii were not accompanied by a corresponding increase in cytochrome c oxidase activity (Complex IV, COX), which is typically associated with maximal aerobic capacity in tissues [35]. In contrast, the artificial E124 and natural plant-based VEG dyes significantly increased the activities of complexes I and III. In contrast, the natural animal-derived dye E120 selectively enhanced the activity of Complex III. These results suggest that the functionality or abundance of mitochondrial complexes may be affected differently depending on the type of dye.

The increased proton leak may be attributed to elevated basal proton conductance resulting from ROS-induced oxidative damage [36], whose production is stimulated by all three red food dyes. ROS encompass both free radicals, such as superoxide (O_2_·⁻) and hydroxyl radical (OH·), and non-radical species, including hydrogen peroxide (H_2_O_2_) and peroxynitrite (ONOOH) [13]. These species originate from various intracellular sources, including mitochondria, peroxisomes, lysosomes, the endoplasmic reticulum, and NADPH oxidase complexes [37].

The elevated ROS levels in nauplii exposed to red food dyes may result from direct chemical interactions involving the dye molecules and/or dye-induced stimulation of endogenous ROS-generating pathways. It has been reported that aromatic amines, produced during the degradation of carminic acid, a by-product present in the food dye E120 due to the manufacturing process [38], can generate ROS through redox cycling mechanisms [39]. Additionally, E124 has been shown to elevate ROS levels, consistent with findings that many azo dyes can induce free radical production in both humans and aquatic organisms [40]. The natural dye VEG contains polyphenols [41], which, although typically known for antioxidant properties, can exhibit pro-oxidant effects under certain conditions and concentrations [42], potentially accounting for the observed increase in ROS production. Regarding endogenous sources, the increased activity of NOX observed in this study may contribute to the elevated ROS levels following dye exposure. In crustaceans, NOX plays a key role in the innate immune response by generating superoxide anions and hydrogen peroxide to combat pathogen invasion and exposure to foreign substances [43,44]. Moreover, NOX activation has been proposed as a general adaptive mechanism used by animals to mitigate the toxic effects of environmental pollutants [45].

Recent findings have also demonstrated that NOX in the shrimp *Litopenaeus vannamei* plays a crucial role in the immune response by regulating the expression of antioxidant genes [46]. Specifically, RNA interference-mediated silencing of NOX significantly reduced both ROS production and the expression levels of key antioxidant enzymes, SOD and CAT, underscoring a dual role for NOX in both ROS generation and the modulation of antioxidant defence mechanisms [46].

In our experiments, exposure to the different dyes elicited variable effects on the activities of antioxidant enzymes. Specifically, both E120 and E124 increased the activities of GPX and GR, whereas the VEG dye enhanced only GR activity. CAT and GPX are involved in the degradation of H_2_O_2_ [47]; however, their functional roles differ in substrate affinity. GPX has a higher affinity for H_2_O_2_ than CAT and is therefore more effective at scavenging H_2_O_2_ at lower concentrations [48]. Consequently, in our study, the observed increase in E120 and E124 GPX activity alone may be sufficient to counterbalance the relatively modest rise in ROS levels.

Cellular GR activity is essential for maintaining intracellular redox balance by converting oxidised glutathione (GSSG), produced during the GPX-catalysed reduction of H_2_O_2_, back into its reduced form (GSH). This recycling process sustains the GSH/GSSG ratio, a critical determinant of redox homeostasis. The observed increase in GR activity suggests that the enzyme’s capacity to regenerate GSH remains intact under dye exposure [49].

Notably, only E120 led to an increase in activity of SOD, which catalyses the dismutation of superoxide radicals into hydrogen peroxide and molecular oxygen [50].

The different effects of the dyes on antioxidant enzyme activity could depend on their different capacity to modulate antioxidant enzymes regulated by multiple factors that can affect their activity or antioxidant gene expression [51]. Our data do not allow us to define the factors involved in the observed effects. In nauplii hatched in the presence of the VEG dye, the situation is further complicated by the presence of components with unknown concentrations, such as maltodextrin and citric acid, that seem to be able to increase the activity of antioxidant enzymes [52,53]. On the contrary, we only found an increase in GR. Furthermore, total antioxidant capacities were not increased in VEG, suggesting that, under our conditions, polyphenols’ pro-oxidant and antioxidant activities are balanced.

While antioxidant enzymes play a critical role in the cellular defence against oxidative stress, evaluating their activity alone may provide an incomplete picture of the organism’s antioxidant capacity. Indeed, increased enzymatic activity may reflect a compensatory response to elevated oxidative stress rather than enhancing the overall antioxidant defence system [54]. We also measured total antioxidant capacity to better understand the dyes’ effects on Artemia nauplii. Our findings revealed a significant increase in total antioxidant capacity in nauplii homogenates following exposure to E120 and E124, whereas no significant change was observed in the VEG-treated group.

To gain information about oxidative stress, we measured markers of protein and lipid oxidative damage. Surprisingly, the increase in protein oxidative damage, measured as protein carbonyl content, was found to increase only in nauplii exposed to E124 despite increased enzymatic antioxidant activity and total antioxidant capacity.

Regarding lipid oxidative damage, we measured two markers: lipid hydroperoxides (HPs) and malondialdehyde (measured as TBARs) as markers of the early and late phases of the lipid peroxidation process [55]. Despite the ROS content increase, HPs decreased significantly in nauplii exposed to E120 and E124 dyes and, to a lesser extent, in those exposed to VEG dye. HP are the primary compounds derived from ROS-induced lipid oxidation, and that is why they are used as early markers of lipid oxidative damage resulting from increased ROS levels. However, HPs are unstable and can be metabolised by GPX or decompose into more stable but still reactive and potentially toxic secondary compounds, such as alkanes, aldehydes, ketones, alcohols, and furans [56]. Interestingly, the marked reduction in HP levels in E120 and E124 agrees with the marked increase in their GPX activities, while in VEG, the lower reduction in HP level agrees with the lack of change in GPX activity that is only partially compensated by the increase in GR activity.

Surprisingly, we found that the levels of TBARs, which are correlated to malondialdehyde content, increase in nauplii hatched in the presence of the red food dyes studied, with a more pronounced effect for E124. We cannot exclude the fact that the increased content of MDA can depend on reduced activity or the content of the mitochondrial enzyme aldehyde dehydrogenase. Indeed, it has been found that a probable biochemical route for MDA metabolism involves its oxidation by mitochondrial aldehyde dehydrogenase, followed by decarboxylation to produce acetaldehyde, which is oxidised by aldehyde dehydrogenase to acetate and further to CO_2_ and H_2_O [55]. Some environmental pollutants can reduce the activity of aldehyde dehydrogenase [57], and it is plausible that the increase in MDA in our experiment may be due to the enzyme’s inefficiency.

## 5. Conclusions and Limitations of the Study

The overall results reported in this study suggest that the tested red food dyes are detrimental to nauplii populations of *Artemia franciscana*. Oxygen consumption is increased by natural dyes E120 and VEG, suggesting alterations in the mitochondrial compartment. The analysis of mitochondrial complex activities confirms such a hypothesis and indicates that such alterations are also verified in E124-exposed nauplii without changes in respiratory capacity. Furthermore, although all dyes increase ROS content, malondialdehyde (the late marker of lipid oxidative damage) measured as TBARs and carbonyl (the marker of protein oxidative damage) content are strongly increased owing to E124 exposure, notwithstanding the increase in antioxidant enzyme activity and TAC. In nauplii exposed to natural dyes (E120 and VEG), while TBARs are comparable, the activities of most antioxidant enzymes and TAC increase in E120 but not in VEG. Therefore, our data indicate that among the tested red dyes, E124 is the most harmful. Furthermore, our data support the observation that red dyes alter the development processes [32] and underline that their use and discharge into water must be addressed.

However, it should be noted that this study has limitations that do not allow for a fully exhaustive explanation of some of the observed results. First, the study was conducted on naupliar populations whose composition in terms of larval stages was not investigated in our experimental conditions. Changes in the ratios between the various larval stages may be responsible for the observed effects. However, it should be emphasised that analysing the parameters we investigated is challenging to perform by separating the various larval stages.

Furthermore, a dose-response study would have allowed us to better understand the mechanisms of response to the dyes. However, such a study would require knowledge of the environmental concentrations of red food dyes, which is not currently available.

Furthermore, since our results suggest alterations affecting the mitochondrial compartment, it would have been interesting to investigate the functionality of isolated mitochondria and mitochondrial dynamics.

Finally, conducting studies on aerobic and anaerobic energy production and cellular energy allocation would be interesting to gain insight into the amount of energy needed for detoxification and consequently not used for other physiological processes.

## Figures and Tables

**Table 1:** Main red molecules in each dye used.

Name	Colourant	Chemical Structure
E124	Ponceau red	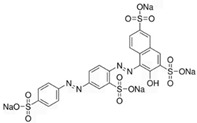
E120 Cochineal red	Carminic acid	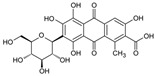
Red Vegan (VEG) from radish, black currant and apple extracts	Pelargonidin (red radish) [17] Cyanidin (Black currant and apple) [17]	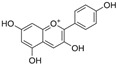 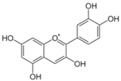

## Data Availability

Data will be made available on request.
